# Sperm Motility Evaluation in Stallion Fresh, Cooled and Frozen Semen Using a Portable Computer‐Assisted Sperm Analysis System

**DOI:** 10.1111/rda.70052

**Published:** 2025-03-20

**Authors:** Leonardo F. C. Brito

**Affiliations:** ^1^ Department of Clinical Studies, New Bolton Center University of Pennsylvania School of Veterinary Medicine Kennett Pennsylvania USA

**Keywords:** CASA, computer‐assisted sperm analysis, fertility, semen, sperm motility, stallion

## Abstract

Semen analysis is an important laboratory diagnostic test for stallions. Evaluation of sperm motility is integral to basic semen analysis and results are important for breeding management and clinical practice. Computer‐assisted sperm analysis (CASA) allows objective sperm motility evaluation and increases analytical precision. The objective of the present study was to validate a portable CASA system (AndroScope). Fresh/cooled semen samples (33 ejaculates, 18 stallions) and frozen semen (40 ejaculates and one epididymal flush, 27 stallions) were evaluated using the AndroScope and the IVOS II CASA system (‘gold standard’). Positive associations (R^2^ = 0.31 to 0.83, *p* < 0.0001) between the systems were observed for all sperm motility estimates. The percentage of motile sperm was lower (*p* < 0.0001) with the AndroScope than with IVOS II (mean difference: −4.2%), but the percentage of progressively normal sperm did not differ between the two systems. Systemic differences were observed for sperm kinetic estimates. Sperm velocities, ALH, and BCF were lower (*p* < 0.0001) with the AndroScope, observations likely associated with the slower video acquisition frame rate used by this system when compared with the IVOS II (45 Hz vs. 60 Hz, respectively). In conclusion, the AndroScope produced precise results for all sperm motility parameters. The ability to identify and track sperm, and to analyse single sperm kinematics seemed similar to that of the ‘gold standard’ system used for validation. The AndroScope video acquisition frame rate must be taken into consideration when evaluating sperm kinetics and setting thresholds for classification of different motile sperm subpopulations.

## 1 Introduction

Semen analysis is the most important laboratory test available for the evaluation of stallion fertility and for the production of adequate samples for artificial in. Evaluation of sperm motility is integral to the basic semen analysis and results are used to attest breeding soundness, diagnose specific causes of subfertility/infertility, guide clinical and management decisions, direct extension rates during the preparation of cooled and frozen semen, and qualify (quality control) semen for specific applications (e.g., artificial insemination, in vitro fertilisation or intracytoplasmic sperm injection). Stallion sperm motility is commonly evaluated subjectively, with the operator assigning a percentage to a sample after observation under a microscope for a few seconds. Proper evaluation requires mostly a microscope equipped with phase‐contrast or differential interference contrast optics at 100–400× magnification with a temperature‐controlled stage (Brito 2024). Subjective evaluation is simple and cost‐effective but challenging to train and standardise. Several studies have reported coefficients of variation (CV) > 15% for subjective sperm motility results among operators (summarised in (Peter et al. 2021)). In a multi‐laboratory study with bovine frozen semen, intra‐laboratory CV ranged from 20% to 55% when sperm motility was evaluated subjectively (Brito 2016).

Objective evaluation of sperm motility has been made possible by the development of computer‐assisted sperm analysis (CASA) systems, which greatly reduce variation in sperm motility results and allow better discrimination of samples than subjective evaluation (Brito 2016; Farrell et al. 1998; Verstegen et al. 2002). The principle of CASA involves capturing video clips of motile sperm using a microscopy setup (hardware) followed by image analysis (software). For image analysis, the system identifies sperm heads and tracks their position in successive frames from the video clips. Therefore, CASA systems generate kinematic data on individual sperm, such as velocity and trajectory pattern. Sperm can then be classified according to their kinematics into subpopulations such as motile or progressively motile sperm (Amann and Waberski 2014; Peter et al. 2021). Although commercial CASA systems have been marketed for almost 40 years, widespread adoption of the technology in equine breeding operations and clinics has been slowed down by equipment cost. Open‐source CASA software (Giaretta et al. 2017) and portable CASA systems (Dini et al. 2019; Moraes et al. 2019) have been developed for the analysis of sperm motility as alternatives to more expensive CASA systems. The AndroScope is a new portable CASA system (hardware 0.65 kg, 14 × 8.5 × 7 cm) with a built‐in heating unit and imaging system for standard microscope slides, including magnifying phase‐contrast lens, high‐speed camera and focus adjustment. Video capture and analysis are performed using proprietary software running on a tablet or laptop connected to the hardware.

CASA results depend on several factors, including system features (e.g., video acquisition frame, sperm tracking algorithm), classification settings (e.g., kinematic thresholds defining progressively motile sperm), sample characteristics (e.g., species, extender, sperm concentration), and sampling procedures (e.g., slide/chamber type, number of fields, number of sperm). Proper validation of each output measure for each system is necessary to ensure accurate and precise results can be generated. Therefore, the objective of the present study was to validate the portable AndroScope CASA system against another CASA system considered to be a ‘gold standard’ under conditions that mimic most of the applications required for stallion semen analysis.

## 2 Materials and Methods

### 2.1 Experimental Design

All procedures involving live animals were performed according to the United States Government Principles for the Utilisation and Care of Vertebrate Animals Used in Testing, Research and Training and were approved by the Institutional Animal Care and Use Committee at the University of Pennsylvania. Semen samples were produced during the clinical routine at the Hofmann Center for Animal Reproduction or as part of various research studies. Most stallions had produced foals before, but reproductive history was unknown in some cases. Fresh/cooled semen samples were prepared using 33 ejaculates from 18 stallions (1–2 ejaculates/stallion), 3 to 29 yearsold, from various breeds (4 American Quarter Horse, 4 Welsh Pony, 3 Trakehner, 2 Arabian, 2 Thoroughbred, 2 Warmblood, Cleveland Bay). Fresh semen samples were evaluated immediately after extension (0 h) and cooled samples were evaluated at 24 h and 48 h after storage at 5°C. Frozen semen samples were prepared using 40 ejaculates and one epididymal flush from 27 stallions (1–2 ejaculates/stallion), 4 to 19 yearsold, from various breeds (7 Arabian, 6 Standardbred, 5 American Quarter Horse, 2 Thoroughbred, 2 Trakehner, Andalusian, Gipsy Vaner, Lusitano, Nokota, Warmblood, Welsh Pony). Frozen semen samples were evaluated at 0 and 30 min after thawing. Semen samples were evaluated side‐by‐side in two replicates using the benchtop HT‐IVOS II (HTCasa II 1.16.311037; Hamilton Thorne Inc., Beverly, MA, USA) and the portable AndroScope (v. 1.1.0.0; Minitube, Tiefenbach, Germany).

### 2.2 Semen Processing

Semen was obtained using a Missouri‐style artificial vagina with the stallion mounted on a dummy. Immediately after collection, semen was filtered to remove gel, and the ejaculate volume was inferred by weight. Sperm concentration was determined using a Model 534B densimeter (Animal Reproduction Systems, Chino, CA) or a NucleoCounter SP100 (ChemoMetec, Allerod, Denmark). Sperm motility was evaluated subjectively using wet preparations and phase‐contrast microscopy (200×). Only samples with > 30% estimated progressively motile sperm were processed further. Sperm morphology was determined by examining 100 sperm using formalin‐fixed wet preparations evaluated under 1000× magnification and immersion oil using differential interference contrast (DIC) microscopy.

For preparation of fresh/cooled samples, semen was extended a minimum of 1:3 (v/v) with pre‐warmed INRA96 extender (IMV Technologies, Maple Grove, MN, USA) to a final concentration of 25 to 50 × 10^6^ sperm/mL. In some cases, semen was extended 1:1 (v/v) with pre‐warmed INRA96 extender and allocated into 50‐mL conical tubes. Then, 1 mL of cushion fluid (MaxiFreeze, IMV; CushionFluid, Minitube, Verona, WI, USA; or OptiPrep, Sigma‐Aldrich, St. Louis, MO, USA) was layered beneath the semen, and tubes were centrifuged at 1000× g for 20 min at room temperature. After centrifugation, the cushion and supernatant were removed and discarded. Sperm pellets were resuspended at room temperature to a concentration of 50 × 10^6^ sperm/mL using INRA96 extender. For cooling, samples were stored at 5°C for 48 h in an Equitainer (HAMILTON BIOVET; Ipswich, MA, USA).

For preparation of frozen samples, semen was cushion centrifuged as described above. Sperm pellets were then resuspended at room temperature to 150–250 × 10^6^ sperm/mL using BotuCRIO extender (BotuPharma, Phoenix, AZ, USA) and packaged into 0.5‐mL straws at room temperature. Straws were frozen in a programmable freezer (Micro Digitcool; IMV Technologies) with 20 min at 5°C, then 5°C to −120°C at 40°C/min. Alternatively, straws were refrigerated at 5°C for 20 min, then layered 5 cm over liquid nitrogen for 15 min. Regardless of freezing method, straws were ultimately plunged and stored in liquid nitrogen.

### 2.3 Semen Evaluation

All analyses were performed by the same operator. Fresh semen samples were evaluated on the day of semen collection immediately after initial semen extension (0 h), whereas cooled samples were evaluated after 24 h and 48 h of cold storage in the Equitainer. Prior to analysis, semen sample aliquots were added to pre‐warmed INRA96 extender to produce 1 mL containing 20–25 × 10^6^ sperm/mL and were incubated at 38°C for 10 min. Frozen straws were thawed at 37°C for 45 s, and the contents were transferred into a pre‐warmed tube. Prior to analysis, aliquots of frozen semen samples were added to pre‐warmed BotuCRIO extender to produce 1 mL containing 20–25 × 10^6^ sperm/mL. Sperm motility was evaluated immediately after dilution and after 30 min of incubation at 38°C. For analysis, 3.3 μL of the sample was loaded into one chamber of a pre‐warmed Leja slide (4 chambers, 20 μm; IMV Technologies) and the loading port was lightly touched with absorbable paper to eliminate sperm drift in the sample. An analysis was obtained using either the IVOS II or the AndroScope; then, another chamber of the Leja slide was loaded, and another analysis was obtained using the other system. The procedure was repeated so that two analyses (two chambers) were obtained for each system. The order in which the equipment was used was alternated across samples. The sample was maintained in a warming block throughout the process and completing the analyses (two chambers for each equipment) took approximately 5 min.

For the IVOS II, analysis settings used in our clinical practice were selected. After verifying that illumination was acceptable and the sample was focused, videos were acquired at 37°C at a frame rate of 60 Hz, with 45 frames used for analysis. Six fields were evaluated in each chamber using the automated stage and sequential auto‐capture mode. Sperm detection parameters included 110 minimum brightness, head size 10–50 μm^2^, 1%–90% elongation, and enabled tail detection for identification of static sperm. For the AndroScope, the ‘Equine’ profile was selected for analysis (hardware specifications and settings were provided by the manufacturer; personal communication). Six fields were evaluated in each chamber by manually positioning the slide into the slide insertion area of the hardware. The camera offset X and Y were adjusted so that the focus center was in the middle of the analysis image. The threshold was adjusted until the binary display showed sperm heads and tail attachment. After verifying that the sample was focused, videos were acquired at 37°C at a frame rate of 45 Hz, with 45 frames used for analysis.

Screen playback was used to determine that single sperm detection and tracking appeared correct prior to saving the results. Parameters evaluated during the analysis included percent total motility, percent progressive motility, curvilinear velocity (VCL), average path velocity (VAP), straight‐line velocity (VSL), straightness (STR), amplitude of lateral head displacement (ALH) and beat cross frequency (BCF) (Peter et al. 2021). For the IVOS II, sperm were considered motile when VAP was > 4 μm/s and VSL was > 1 μm/s, and considered progressively motile when VAP was > 30 μm/s and STR was > 50%. For the AndroScope, sperm were considered motile when ALH > 4 μm or BCF > 4 Hz, and considered progressively motile when VCL was > 40 μm/s and VSL was > 10 μm/s. Separate sperm kinetics data were generated for motile and progressively motile sperm subpopulations.

### 2.4 Statistical Analysis

Statistical analysis was conducted using GraphPad Prism V. 9.4.0 (GraphPad Software, San Diego, CA, USA) and Statistix 8 (Analytical Software, Tallahassee, FL, USA). Coefficients of variation (CV) were calculated based on the replicate samplings (two chambers). Replicate results were averaged, and the agreement of results obtained by the two pieces of equipment was determined by linear regression and through evaluation of Bland–Altman plots. The effect of the CASA system on sperm motility parameters and on the intra‐sample CV was determined using a paired t‐test.

## 3 Results

**FIGURE 1:**
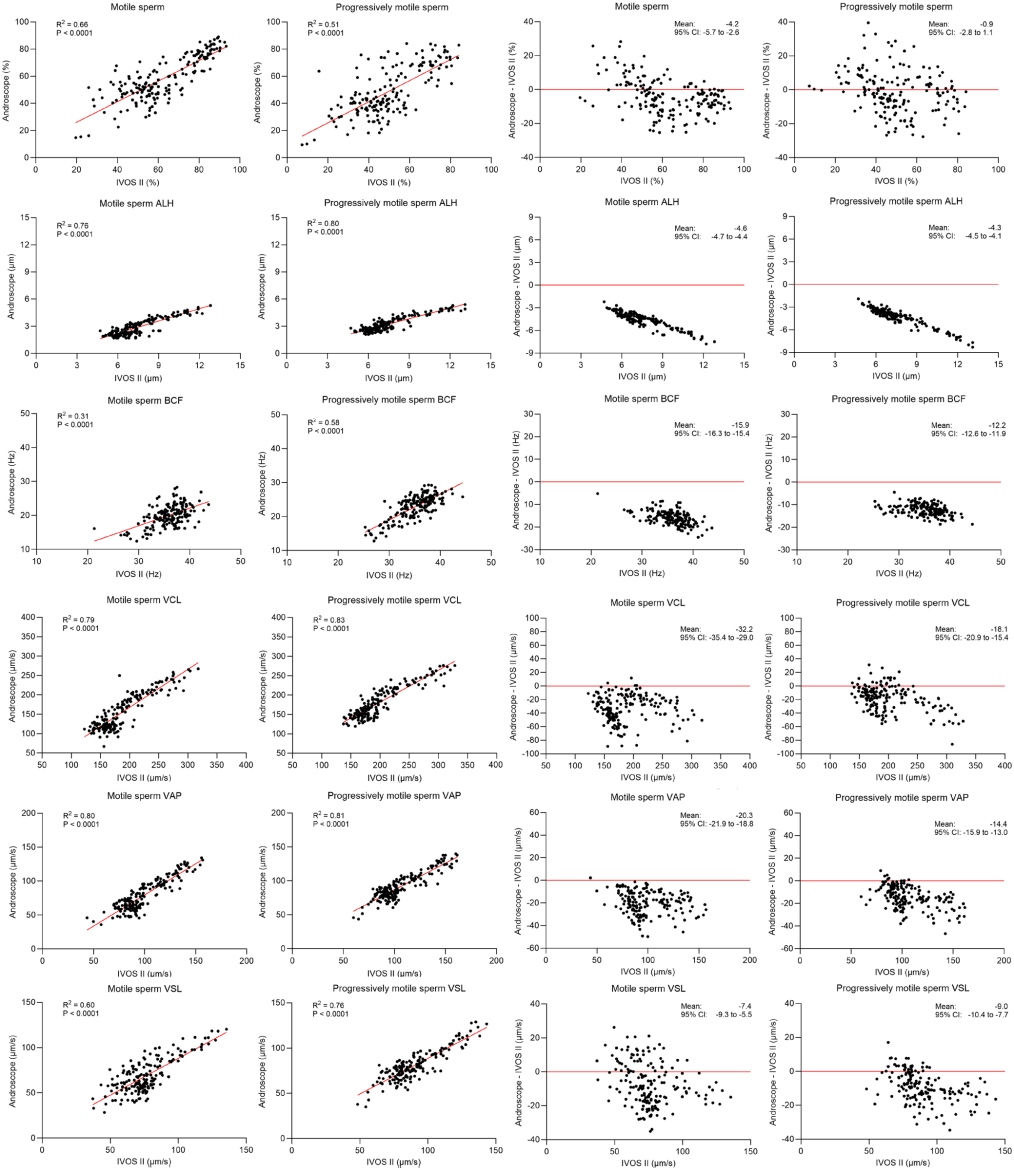
Linear regressions (two graphs on the left) and Bland–Altman plots (two graphs on the right) for stallion sperm motility parameters in 174 semen samples (33 fresh, 63 cooled, and 78 frozen) obtained using the HT‐IVOS II or the Minitube AndroScope. ALH: amplitude of lateral head displacement, BCF: beat cross frequency, STR: straightness, VAP: average path velocity, VCL: curvilinear velocity, VSL: straight line velocity.

Results of initial ejaculate analysis are included as supporting Information S1. The final data analysis included a total of 174 samples (33 fresh, 63 cooled, and 78 frozen) and 348 replicates per system. Linear regression showed significant (*p* < 0.0001) positive associations between results obtained with the IVOS II and the AndroScope (Figure 1). There were moderate (R^2^ = 0.51 to 0.66) associations for the percentages of motile and progressively motile sperm, motile sperm VCL, and progressively motile sperm BCF. There was a good association for all other sperm kinematics (R^2^ = 0.76 to 0.83). The total number of evaluated sperm was greater (*p* < 0.0001) with the AndroScope. Progressive and progressive STR did not differ between systems, but all other parameters were lower (*p* < 0.0001) with the AndroScope than with the IVOS II (Table 1). Bland–Altman plots revealed that AndroScope results systematically underestimated all sperm motility parameters when compared with the IVOS II (Figure 1). This was particularly evident for ALH, BFC, VCL and VAP. On average, the percentage of motile sperm obtained with the AndroScope was 4.2% lower than the percentage obtained with the IVOS II.

**TABLE 1:** Mean (± SEM) stallion sperm motility parameters obtained using the HM‐IVOS II or the Minitube AndroScope for the analysis of 174 semen samples (33 fresh, 63 cooled, and 78 frozen).

	IVOS II	AndroScope
TOTAL SPERM (#)	431 ± 8^a^	1102 ± 20^b^
MOTILE (%)	62.3 ± 1.3^a^	58.2 ± 1.2^b^
PROGRESSIVE (%)	49.6 ± 1.2	48.7 ± 1.3
MOTILE ALH (μm)	7.6 ± 0.2^a^	2.9 ± 0.1^b^
MOTILE BCF (Hz)	35.9 ± 0.3^a^	20.0 ± 0.2^b^
MOTSTR (%)	77.6 ± 0.9^a^	81.6 ± 0.4^b^
MOTILVAP (μm/s)	99.5 ± 1.7^a^	79.2 ± 1.7^b^
MOTILE VCL (μm/s)	189.9 ± 3.2^a^	157.7 ± 3.6^b^
MOTILE VSL (μm/s)	76.3 ± 1.4^a^	68.9 ± 1.5^b^
PROGRESSIVE ALH (μm)	7.6 ± 0.1^a^	3.3 ± 0.1^b^
PROGRESSIVE BCF (Hz)	35.0 ± 0.3^a^	22.8 ± 0.3^b^
PROGRESSIVE STR (%)	84.5 ± 0.4	84.8 ± 0.4
PROGRESSIVE VAP (μm/s)	106.4 ± 1.7^a^	92.0 ± 1.5^b^
PROGRESSIVE VCL (μm/s)	199.3 ± 3.3^a^	181.2 ± 3.0^b^
PROGRESSIVE VSL (μm/s)	89.5 ± 1.4^a^	80.5 ± 1.3^b^

*Note:* ^a,b^Columns with different superscripts differ (*p* < 0.0001).

Abbreviations: ALH: amplitude of lateral head displacement, BCF: beat cross frequency, STR: straightness, VAP: average path velocity, VCL: curvilinear velocity, VSL: straight‐line velocity.

There were small but significant differences in CV between systems (Table 2). When compared to the IVOS II, the AndroScope had lower (*p* < 0.05) CV for total, motile STR, progressive ALH, and progressive VCL, but had a greater (*p* < 0.05) CV for motile, motile BCF, motile VCL, motile VAP, and motile VSL. Overall, the CV for estimates of motile and progressively motile sperm were < 8%, whereas the CV for estimates of sperm kinetics was < 5%, regardless of thesystem.

**TABLE 2:** Mean (± SEM) coefficients of variation (CV, %) based on duplicate samplings for various parameters of stallion sperm motility analysis obtained using the HT‐IVOS II or the Minitube AndroScope for the analysis of 174 semen samples (33 fresh, 63 cooled, and 78 frozen).

	IVOS II	AndroScope
TOTAL SPERM	7.8 ± 0.6^a^	6.2 ± 0.4^b^
MOTILE	5.9 ± 0.4^a^	7.0 ± 0.5^b^
PROGRESSIVE	7.3 ± 0.5	6.9 ± 0.4
MOTILE ALH	3.1 ± 0.2	3.4 ± 0.3
MOTILE BCF	2.1 ± 0.1^a^	3.6 ± 0.3^b^
MOTILE STR	2.0 ± 0.1^a^	1.3 ± 0.1^b^
MOTILE VAP	3.4 ± 0.3^a^	4.3 ± 0.3^b^
MOTILE VCL	3.1 ± 0.2^a^	3.7 ± 0.3^b^
MOTILE VSL	3.6 ± 0.3^a^	4.7 ± 0.3^b^
PROGRESSIVE ALH	3.2 ± 0.2^a^	2.0 ± 0.1^b^
PROGRESSIVE BCF	2.1 ± 0.1	2.3 ± 0.1
PROGRESSIVE STR	1.1 ± 0.1	1.0 ± 0.1
PROGRESSIVE VAP	2.5 ± 0.2	2.4 ± 0.1
PROGRESSIVE VCL	2.8 ± 0.2^a^	1.9 ± 0.1^b^
PROGRESSIVE VSL	2.7 ± 0.2	2.8 ± 0.2

*Note:* ^a,b^Columns with different superscripts differ (*p* < 0.05).

Abbreviations: ALH:amplitude of lateral head displacement, BCF: beat cross frequency, STR: straightness, VAP: average path velocity, VCL: curvilinear velocity, VSL: straight‐line velocity.

## 4 Discussion

Although different CASA systems are based on similar principles, there are several differences in the hardware and software among different systems. Therefore, validation of each output measure for each system is necessary before one can be sure that data serving as the basis for breeding management, clinical practice, and R&D decisions are accurate and precise (Amann and Waberski 2014; Brito 2024). The present validation study included a representative population of stallions of various breeds and a wide range of sperm motilities. Not only the proportion of motile sperm might differ among fresh, cooled, and frozen semen, but sperm kinetics might also differ depending on the sample type (Yeste et al. 2018); therefore, the use of different sample types for validation was also important. Hamilton Thorne pioneered the design of CASA equipment and launched the first commercial system for stallion semen analysis in 1986. The IVOS system was launched in 1992 and is periodically updated. The benchtop HT‐IVOS II is the most recent iteration of the equipment and is widely used for clinical and research applications in various species (Amann and Waberski 2014; Mortimer et al. 2015). For these reasons, the IVOS II was selected as the ‘gold standard’ for AndroScope comparison.

Validation of analytical results requires determining their accuracy and precision (Brito 2024). Accuracy refers to the agreement between the test result and the ‘true value’. Precision is determined by comparing test results from repeated measurements on the same sample. The coefficient of variation was used to determine random error and estimate precision. Although significant differences between systems were observed in the CV for some parameters, both systems were considered very precise (CV ranged from 1% to 7%), similar to the reported intra‐technician CV for CASA sperm motility reported in most studies (< 10%; Peter et al. 2021). Linear regression analysis helped establish the strength of the association between the two systems. All sperm motility parameters analysed showed moderate or good positive associations, suggesting that the systems have a similar ability to track and analyse individual sperm.

Pairwise comparisons and Bland‐Altman plots were used to measure systematic errors and test accuracy. The systemic differences observed in all sperm kinetic parameters were likely the result of the slower video acquisition frame rate used by the AndroScope (45 Hz) when compared with the IVOS II (60 Hz). Acquisition frame rate is important because it directly impacts the shape of the reconstructed sperm trajectory. Stallion sperm velocity determined using CASA increases with increasing acquisition frame rate up to approximately 250 Hz (Gacem et al. 2020) and most authors recommend using a minimum of 50–60 Hz (Amann and Waberski 2014; Mortimer et al. 2015). Similarly to what was observed in the present study, the effect of frame rate has been shown to be more pronounced for VCL and VAP, which are calculated based on the entire sperm trajectory, rather than for VSL, which is calculated based only on the sperm location in the first and last frames (Bompart et al. 2018). The systemic differences observed for ALH and BFC were particularly pronounced, suggesting that meaningful differences might also exist in how the systems' algorithms calculate these variables. In addition, ALH was the only parameter for which the difference did not seem randomly distributed but rather decreased linearly with increasing IVOS II estimates.

The percentage of motile sperm was significantly lower with the AndroScope, but the mean difference was relatively small. In addition, the percentage of progressively motile sperm did not differ between the two systems. Differences between the two systems in the algorithms used to classify sperm into different subpopulations according to motility included both different variables used in the model and different thresholds for those variables. Therefore, it seems that the model used by the AndroScope (‘Equine profile’) accounted to a certain degree for the overall lower velocities obtained with this system. CASA systems must always accompany documentation of hardware and software specifications required for informed, educated users to interpret the results. In addition, since hardware and software specifications have demonstrable effects on sperm motility estimates, reputable scientific journals require researchers to provide full details and settings of CASA systems used in research as essential for study reproducibility.

Sperm concentration and slide/chamber type are important sources of CASA variation. Precise and accurate results can be obtained with concentrations between 2 and 50 × 10^6^ sperm/mL (Amann and Waberski 2014; Yeste et al. 2018). Sperm collisions during video acquisition may result in errors during the evaluation of individual tracks, since the algorithm may not be able to properly and continuously identify sperm heads in the frames immediately after the collision. These errors are minimal with sperm concentration < 30 million/mL but make results obtained with concentrations greater than 50 × 10^6^ sperm/mL practically meaningless. The chamber depth, shape, and loading method might affect the ability of sperm to move unrestricted and the sperm distribution within the chamber. Small chamber depth might restrict the natural helical sperm movement, whereas large depth hampers individual sperm tracking as sperm are likely to swim up or down and thus be out of the field of view (Bompart et al. 2018; Hoogewijs et al. 2012). Capillary loaded, disposable chambers with 20 μm depth, such as the Leja slides used in the present study, are considered ideal (Amann and Waberski 2014). When the IVOS II was used to evaluate stallion frozen semen samples, the percentages of motile and progressively motile sperm and VCL did not differ among samples at 10, 30, or 50 × 10^6^ sperm/mL (Hernández‐Avilés et al. 2024).

The variation in sampling associated with field selection can be minimised in the IVOS II by using the automated stage and sequential auto‐capture mode. These features ensure that evaluations are always performed at the same locations of the chamber, improving sampling standardisation. Operator bias is a common challenge with manual field selection, as fields with apparently lesser motile sperm tend to be ignored in favour of fields with greater motile sperm (Brito 2024). In the present study, the total number of fields evaluated in each chamber was set to six so that the same number could be used for both equipment. Due to lower imaging magnification, six was the maximum number of non‐overlapping fields that could be captured with the 4‐chamber Leja slide when using the AndroScope. This also eliminated field selection bias since all imageable fields were analysed. The variation in sampling created by selecting a fixed number of sperm for analysis is a random error referred to as the statistical sampling error. When sperm motility is evaluated, a minimum of 400 sperm should be included in the analysis in order to minimise statistical sampling error (Brito 2024; WHO 2021). Since the total sperm number for the IVOS II was above 400, the sampling error difference was minimal even with the 2.5× greater total sperm number observed with the AndroScope.

Different total sperm numbers reflect the different imaging systems used in the two systems. The IVOS II utilises a 10× negative‐phase contrast objective lens, whereas the AndroScope utilises a lower magnification positive‐contrast lens. The magnification at which AndroScope analysis is performed is not stated in technical documents, and a direct relationship might not be completely accurate since the size of the camera field of view might also differ between equipment; but considering total sperm counts, the magnification utilised by the AndroScope was 39% of that utilised by the IVOS II. CASA systems might produce precise estimates of sperm concentration, as would have been the case in the present study, since CV for total sperm number was < 8% for both systems. However, CASA accuracy when evaluating sperm concentration is marred by several technical issues and variations, and for these reasons, CASA use is not currently recommended for sperm concentration analysis (Brito et al. 2016; WHO 2021).

Different imaging magnification also precluded a comparison of the systems' abilities to identify and differentiate sperm from other particles. Total sperm numbers were consistently greater in frozen than in fresh and cooled semen samples regardless of equipment (data not shown), suggesting that a larger number of inert particles might have been incorrectly identified as sperm with BotuCrio extender than with INRA96 extender. This was also the subjective impression when using the on‐screen playback with colour‐coded objects option available with both systems. INRA96 contains milk phosphocaseinate, which appears as fine particles when observed under phase‐contrast microscopy and are only occasionally incorrectly classified as sperm. BotuCrio is a freezing extender that, in addition to cryoprotectants, also contains egg yolk and skim‐milk proteins; therefore, this extender contains more larger particles that can be misidentified as sperm. Frozen semen was diluted with BotuCrio prior to analysis of frozen semen in this study so that samples in extenders with different compositions and viscosities could be evaluated, but in practice, the use of clearer extenders for dilution is preferable to minimise the presence of particles that can interfere with CASA results.

The operator is an important source of CASA variation. An operator who does not understand the equipment, the principles of the analysis, is not adequately prepared to judge whether the results obtained are optimal, and simply accepts the results generated by equipment without critical thinking misses any and all benefits that CASA technology offers. Use of standardised operating procedures and operator training are essential for accurate and precise CASA results (Brito 2024). E‐learning methods addressing standardisation of sample preparation and analysis with CASA, including text, pictures, and videos, as well as the most probable sources of error, have been shown to decrease operator variability (Ehlers et al. 2011). General guidelines of best practices for training and standardisation of sperm motility analysis procedures can be found elsewhere (Brito 2024; Peter et al. 2021).

In conclusion, the portable AndroScope CASA system is a viable alternative for the analysis of fresh, cooled, and frozen stallion semen. Precise results were obtained for all analysed parameters, including percentages of motile and progressively motile sperm and sperm kinematics. The ability to identify and track sperm, as indicated by sperm kinematic characteristics, seemed similar to that of the ‘gold standard’ system used for comparison. The AndroScope acquisition frame rate should be taken into consideration when evaluating sperm kinetics. The effects of different threshold settings (profiles) for the evaluation of different semen sample types should also be investigated.

## Author Contributions

The author takes full responsibility for this article.

## Conflicts of Interest

The author declares no conflicts of interest.

## Supporting information


Appendix S1.

## Data Availability

The data that support the findings of this study are available on request from the corresponding author. The data are not publicly available due to privacy or ethical restrictions.
