# Supplementary material for: Sperm Motility Evaluation in Stallion Fresh, Cooled and Frozen Semen Using a Portable Computer‐Assisted Sperm Analysis System
Source: Reprod Domest Anim. 2025 Mar 20;60(3):e70052. doi: 10.1111/rda.70052 (PMC11924987; doi:10.1111/rda.70052)
Supplement: Supplementary file 1 — Appendix S1. [file RDA-60-e70052-s001.pdf]

Table S1. Characteristics of the ejaculates used for production of fresh and cooled semen samples.

Stallion	Breed	Age	VOL	CC	NOR	ACR	HD	DT	PD	DD	BMP	OMP	BCT
		y	mL	x 10^6/mL	%	%	%	%	%	%	%	%	%
Stallion 1	AQH	8	19	485	80	1	4	2	8	0	6	3	0
Stallion 1	AQH	8	27	378	74	0	0	6	5	0	4	1	0
Stallion 2	AQH	10	51	280	45	0	30	2	24	2	1	7	7
Stallion 2	AQH	10	38	208	75	0	1	3	7	3	5	5	1
Stallion 3	AQH	17	77	119	81	0	1	0	6	0	1	0	2
Stallion 3	AQH	17	83	197	64	0	10	0	17	7	4	1	1
Stallion 4	AQH	23	53	248	30	0	8	2	46	0	2	12	6
Stallion 4	AQH	23	49	511	33	0	13	1	53	1	4	3	2
Stallion 5	Arabian	9	22	505	64	0	3	0	10	11	10	2	1
Stallion 5	Arabian	9	27	477	60	1	1	0	8	16	12	1	2
Stallion 6	Arabian	20	19	214	31	0	4	1	11	2	41	6	6
Stallion 6	Arabian	20	83	138	34	0	6	0	20	0	24	6	14
Stallion 7	Cleveland Bay	5	17	102	41	5	11	1	6	5	14	18	8
Stallion 7	Cleveland Bay	5	37	83	31	8	4	1	7	2	20	22	10
Stallion 8	Thoroughbred	9	58	69	54	1	32	0	4	1	13	4	2
Stallion 8	Thoroughbred	9	78	131	36	2	44	2	10	4	14	4	4
Stallion 9	Thoroughbred	11	110	102	60	3	3	1	12	8	13	1	1
Stallion 10	Trakehner	5	37	282	28	5	12	2	15	2	17	19	12
Stallion 11	Trakehner	8	41	668	79	2	4	3	1	1	5	5	3
Stallion 11	Trakehner	8	87	150	83	0	5	1	1	0	5	3	4
Stallion 12	Trakehner	8	56	228	49	3	19	0	6	2	16	9	4
Stallion 12	Trakehner	8	36	393	45	9	5	4	9	3	12	10	9
Stallion 13	Warmblood	3	93	191	40	7	12	10	9	2	19	5	4
Stallion 13	Warmblood	3	48	129	43	8	5	8	11	2	16	15	2
Stallion 14	Warmblood	3	53	60	40	1	35	1	19	4	4	6	8
Stallion 14	Warmblood	3	21	372	22	3	19	3	21	21	10	10	5
Stallion 15	Welsh Pony	3	10	585	65	1	4	0	9	6	9	7	3
Stallion 15	Welsh Pony	3	13	197	54	3	5	2	23	12	2	3	1

Table S1. Continued...characteristics of the ejaculates used for production of fresh and cooled semen samples.

Stallion 16	Welsh Pony	12	39	237	64	0	8	1	6	2	6	16	1
Stallion 16	Welsh Pony	12	15	313	67	0	6	0	1	1	11	15	1
Stallion 17	Welsh Pony	22	68	229	50	8	9	1	8	6	18	4	5
Stallion 18	Welsh Pony	29	50	136	60	2	7	0	24	1	5	2	0
Stallion 18	Welsh Pony	29	57	137	76	0	12	1	10	0	1	0	3

VOL: volume, CC: sperm concentration, NOR: morphologically normal sperm, ACR: acrosome defects, HD: head defects, DT: detached heads, PD: proximal droplets, DD: distal droplets, BM: bent midpieces, OMP: other midpiece defects, BCT: bent/coiled tails.

Table S2. Characteristics of the ejaculates used for production of frozen semen samples.

Stallion	Breed	Age<br>y	VOL<br>mL	CC<br>x 10^6/mL	TM<br>%	PM<br>%	NOR<br>%	ACR<br>%	HD<br>%	DT<br>%	PD<br>%	DD<br>%	BMP<br>%	OMP<br>%	BCT<br>%
Stallion 1	AQH	8	16	303	89	54	64	3	12	1	9	6	2	2	2
Stallion 2	AQH	10	29	116	80	49	66	3	12		8	0	1	14	0
Stallion 2	AQH	10	33	222	84	67	79	0	9	1	9	0	1	5	1
Stallion 3	AQH	12	32	241	83	60	84	0	10	0	1	1	0	4	0
Stallion 3	AQH	12	37	315	83	65	81	1	7	1	1	0	4	6	0
Stallion 4	AQH	15	25	165	77	55	69	0	10	2	10	2	3	12	1
Stallion 4	AQH	15	13	293	77	59	65	4	7	0	10	3	3	11	2
Stallion 5	AQH	17	72	146	72	51	62	0	16	2	16	1	3	2	0
Stallion 5	AQH	17	26	274	89	74	70	1	17	0	7	4	1	7	1
Stallion 6	Andalusian	15	50	179	80	50	67	0	1	1	11	3	2	3	12
Stallion 6	Andalusian	15	14	392	70	40	58	3	7	3	0	3	3	8	17
Stallion 7	Arabian	7	32	277	97	63	72	0	2	3	2	5	15	4	2
Stallion 7	Arabian	7	89	150	92	58	72	2	2	1	2	0	16	5	0
Stallion 8	Arabian	12	41	148	68	41	52	4	10	15	13	2	1	4	0
Stallion 8	Arabian	12	46	102	84	64	47	4	4	9	17	3	2	8	5
Stallion 9	Arabian	12	52	203	79	69	75	2	3	5	5	4	6	0	8
Stallion 9	Arabian	12	39	132	85	75	80	2	2	3	2	2	3	0	6
Stallion 10	Arabian	18	25	206	69	49	37	1	4	5	11	0	40	4	0
Stallion 10	Arabian	18	8	199	66	52	50	0	8	2	8	0	26	12	0
Stallion 11	Arabian	18	39	150	64	44	38	7	13	2	7	5	18	3	10
Stallion 12	Arabian	19	44	123	81	44	47	1	6	3	8	4	18	17	0
Stallion 13	Gipsy Vaner	16	84	59	70	33	58	1	6	2	14	11	5	3	0
Stallion 13	Gipsy Vaner	16	83	63	73	35	70	2	2	3	7	6	2	5	3
Stallion 14	Lusitano	4	40	227	85	62	56	0	11	2	3	0	12	19	4
Stallion 15	Nokota	4	64	62	76	67	73	0	7	0	9	3	6	7	0
Stallion 16	Standardbred	8	26	646	70	60	72	3	10	1	9	1	6	4	1
Stallion 17	Standardbred	11	43	306	70	50	73	0	10	0	6	4	8	4	1
Stallion 18	Standardbred	17	28	51	70	60	53	3	8	1	15	5	2	9	5
Stallion 19	Standardbred	19	67	500	50	40	39	0	35	1	18	3	13	5	6
Stallion 20	Standardbred	19	67	137	60	50	46	7	5	5	15	1	8	12	1

Table S2. Continued...characteristics of the ejaculates used for production of frozen semen.

Stallion 21	Standardbred	23	97	242	50	40	38	0	24	6	6	11	25	5	0
Stallion 22	Thoroughbred	4	58	202	62	33	63	3	7	4	6	1	5	4	7
Stallion 22	Thoroughbred	4	63	184	69	44	79	0	0	0	4	5	7	0	9
Stallion 23	Thoroughbred	9	59	252	75	43	40	2	38	0	4	15	15	2	7
Stallion 23	Thoroughbred	9	55	91	79	57	44	5	30	1	7	7	16	1	2
Stallion 24	Trakehner	8	13	455	77	62	48	1	20	1	6	2	15	12	8
Stallion 24	Trakehner	8	41	157	78	65	53	3	12	1	5	1	9	13	15
Stallion 25	Trakehner	8	45	197	83	78	77	3	5	2	0	1	5	6	2
Stallion 25	Trakehner	8	87	150	87	81	83	0	5	1	1	0	5	3	4
Stallion 26	Warmblood	5	Epididymal flush		60	40	62	4	3	1	11	6	4	2	8
Stallion 27	Welsh Pony	12	59	132	75	60	75	1	0	5	2	5	4	2	6

VOL: volume, CC: sperm concentration, TM: total sperm motility, PM: progressive sperm motility, NOR: morphologically normal sperm, ACR: acrosome defects, HD: head defects, DT: detached heads, PD: proximal droplets, DD: distal droplets, BM: bent midpieces, OMP: other midpiece defects, BCT: bent/coiled tails.
